# *catena*-Poly[[bis­(*N*,*N*-dimethyl­formamide-κ*O*)zinc]-μ_2_-oxalato-κ^4^ *O* ^1^,*O* ^2^:*O* ^1′^,*O* ^2′^]

**DOI:** 10.1107/S1600536811030479

**Published:** 2011-08-02

**Authors:** Ju Eun Lee, Hong-In Lee

**Affiliations:** a Department of Chemistry and Green-Nano Materials Research Center, Kyungpook National University, Daegu 702-701, Republic of Korea

## Abstract

In the crystal structure of the title compound, [Zn(C_2_O_4_)(C_3_H_7_NO)_2_]_*n*_, the Zn^II^ ion is situated on a twofold rotation axis and has a distorted octa­hedral coordination geometry defined by the O atoms of two dimethyl­formamide mol­ecules and four O atoms of two bidentate oxalate ligands. The oxalate anion is located on an inversion centre and bridges two metal ions, resulting in a polymeric structure with infinite zigzag chains extending parallel to [010].

## Related literature

For related structures, see: Yao *et al.* (2007 ▶); van Albada *et al.* (2004 ▶); Ghosh *et al.* (2004 ▶); Evans & Lin (2001 ▶). For a general review on compounds with metal-organic framework structures, see: Czaja *et al.* (2009 ▶). For the synthesis of the ligand, see: Yoneda *et al.* (1978 ▶).
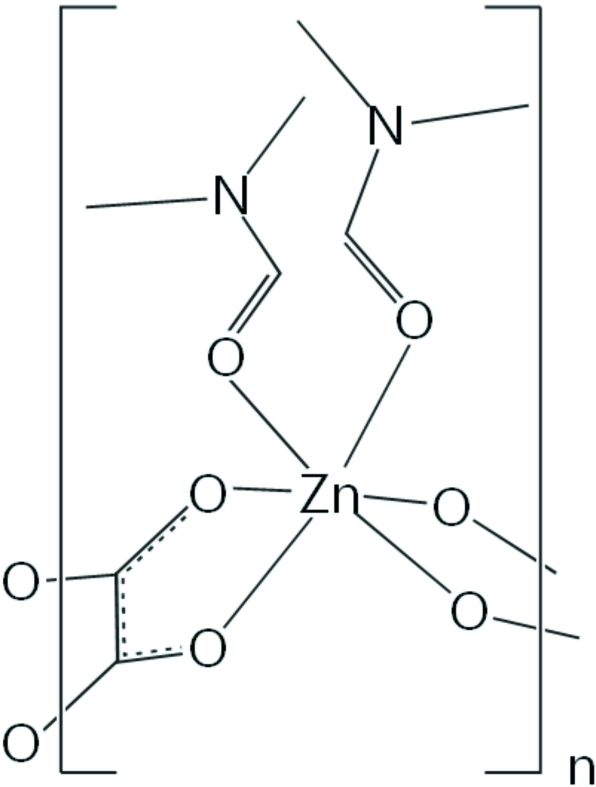


## Experimental

### 

#### Crystal data

[Zn(C_2_O_4_)(C_3_H_7_NO)_2_]
*M* *_r_* = 299.58
Orthorhombic, 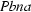

*a* = 7.795 (1) Å
*b* = 9.809 (1) Å
*c* = 15.421 (1) Å
*V* = 1179.1 (2) Å^3^
*Z* = 4
Synchrotron radiation
λ = 0.90000 Å
μ = 2.10 mm^−1^
*T* = 298 K
0.14 × 0.10 × 0.09 mm

#### Data collection

ADSC Quantum210 diffractometer
Absorption correction: multi-scan (*HKL-2000* *SCALEPACK*; Otwinowski & Minor, 1997 ▶) *T*_min_ = 0.757, *T*_max_ = 0.833
839 measured reflections
839 independent reflections
778 reflections with *I* > 2σ(*I*)
θ_max_ = 30.4°

#### Refinement

*R*[*F* ^2^ > 2σ(*F* ^2^)] = 0.058
*wR*(*F* ^2^) = 0.169
*S* = 1.09
839 reflections
81 parameters
H-atom parameters constrained
Δρ_max_ = 0.80 e Å^−3^
Δρ_min_ = −0.82 e Å^−3^

### 

Data collection: *ADSC Quantum-210 ADX* (Arvai & Nielsen, 1983 ▶); cell refinement: *HKL-2000* (Otwinowski & Minor, 1997 ▶); data reduction: *HKL-2000*; program(s) used to solve structure: *SHELXS97* (Sheldrick, 2008 ▶); program(s) used to refine structure: *SHELXL97* (Sheldrick, 2008 ▶); molecular graphics: *CrystalMaker* (CrystalMaker, 2007 ▶); software used to prepare material for publication: *publCIF* (Westrip, 2010 ▶).

## Supplementary Material

Crystal structure: contains datablock(s) I, global. DOI: 10.1107/S1600536811030479/wm2511sup1.cif

Structure factors: contains datablock(s) I. DOI: 10.1107/S1600536811030479/wm2511Isup2.hkl

Additional supplementary materials:  crystallographic information; 3D view; checkCIF report

## Figures and Tables

**Table 1:** Selected bond lengths (Å)

Zn1—O2	2.101 (2)
Zn1—O1	2.104 (2)
Zn1—O3	2.134 (2)

**Fig. 1.**
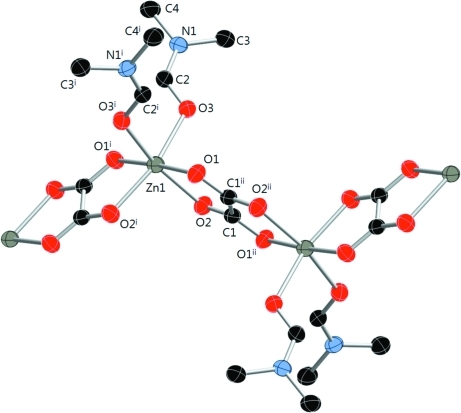
Partial structure of the title compound showing 30% probability displacement ellipsoids and the atom-numbering scheme. All H atoms are omitted for clarity. Symmetry codes: (i) x, -y + 3/2, -z. (ii) -x, -y + 1, -z.

**Fig. 2.**
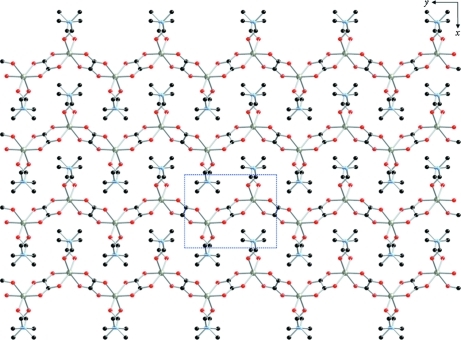
two-dimensional packing structure of the one-dimensional zigzag chains of the title compound viewing along the crystal z-direction (gray, Zn; black, C; red, O; blue, N) Dotted box represents the xy-plane of the unit cell and x-, y- directions are denoted by arrows at upper right corner.

## supplementary crystallographic information

### Comment

Metal-organic frameworks (MOFs) have been widely investigated for their potential and/or practical applications in catalysis, gas storage, and many others fields (Czaja *et al.*, 2009). We aimed at constructing a new functional MOF material using a conducting organic molecule, *viz* tetrathiafulvalene (TTF) functionalized with carboxylate groups, by the hydro(solvo)thermal method. During synthesis, we unexpectedly discovered a Zn^II^-oxalate coordination polymer, (I), forming an infinite one-dimensional zigzag chain. We are currently studying the detailed formation mechanism of the compound.

In the structure of compound (I), the Zn^II^ ion lies on a 2-fold axis and is coordinated by four oxygen atoms of the two bridging oxalate groups and two oxygen atoms of DMF solvent molecules, resulting in a distorted octahedral geometry (Fig. 1). The Zn—O_ox_ bond lengths are in the range of 2.101 (2) - 2.104 (2) Å and the Zn—O_DMF_ bond length is 2.134 (2) Å. The bond angles about the Zn^II^ ion range between 78.62 (8) and 98.81 (9)° for *cis* and between 163.08 (9) and 176.23 (11)° for the *trans* ligands (Table 1). The bond angle of O_ox_—Zn—O_ox_ (78.62 (8)°) is smaller than that of O_DMF_—Zn—O_DMF_ (86.53 (13)°) due to the five-membered chelate ring strain. The Zn—O bond lengths and the bond angles about Zn^II^ are comparable to those of other reported Zn-oxalate coordination polymers (Yao *et al.*, 2007; van Albada *et al.*, 2004; Ghosh *et al.*, 2004; Evans & Lin, 2001). The Zn-oxalate backbone has a zigzag shape with a Zn—Zn—Zn angle of 126.47 (2)° and a Zn—Zn distance of 5.493 (1) Å. The resulting one-dimensional zigzag chains run parallel to [010] and pack effectively through the inter-wedges of the coordinated DMF ligands (Fig. 2).

### Experimental

This experiment was originally intended for synthesis of compounds with metal-organic frameworks, consisting of Zn^II^ ions and tetrathiafulvalene (TTF) functionalized with carboxylate groups [= bis(4-carboxy-1,3-dithiolidene) = 2COOH-TTF]. Bis(4-carboxy-1,3-dithiolidene) was prepared according to literature (Yoneda *et al.* 1978). 2COOH-TTF (0.050 g, 0.17 mmol) and 4,4-bipyridine (0.013 g, 0.098 mmol) were added to 12 ml DMF:H_2_O (5:1, *v*/*v*) solution of [Zn(NO_3_)_2_]^.^6H_2_O (0.051 g, 0.17 mmol) to be stirred for 10 min. The mixture was sealed in a Pyrex test tube and stored at 358 K for 3 days. After cooled down to room temperature, the mixture was filtered and washed with ethanol. Colorless crystals suitable for X-ray analysis were obtained and were dried in air.

### Refinement

All C-bound H atoms were placed in geometrically idealized positions and refined using a riding model with *U*_iso_ = 1.5*U*_eq_ and C–H = 0.96 Å for CH_3_, and *U*_iso_ = 1.2*U*_eq_ and C–H = 0.93 Å for CH.

### Crystal data

**Table:**

[Zn(C_2_O_4_)(C_3_H_7_NO)_2_]	*F*(000) = 616
*M**_r_* = 299.58	*D*_x_ = 1.688 Mg m^−^^3^
Orthorhombic, *P**b**n**a*	Synchrotron radiation, λ = 0.90000 Å
Hall symbol: -P 2ac 2b	Cell parameters from 839 reflections
*a* = 7.795 (1) Å	θ = 5.4–30.4°
*b* = 9.809 (1) Å	µ = 2.10 mm^−^^1^
*c* = 15.421 (1) Å	*T* = 298 K
*V* = 1179.1 (2) Å^3^	Block, colourless
*Z* = 4	0.14 × 0.10 × 0.09 mm

### Data collection

**Table:**

ADSC Quantum210 diffractometer	839 independent reflections
Radiation source: 6BIMX-I synchroton beamlin PLS, KOREA	778 reflections with *I* > 2σ(*I*)
Si111 double crystal	*R*_int_ = 0.000
φ scans	θ_max_ = 30.4°, θ_min_ = 5.4°
Absorption correction: multi-scan (*HKL-2000**SCALEPACK*; Otwinowski & Minor, 1997)	*h* = 0→8
*T*_min_ = 0.757, *T*_max_ = 0.833	*k* = 0→10
839 measured reflections	*l* = 0→16

### Refinement

**Table:**

Refinement on *F*^2^	Secondary atom site location: difference Fourier map
Least-squares matrix: full	Hydrogen site location: inferred from neighbouring sites
*R*[*F*^2^ > 2σ(*F*^2^)] = 0.058	H-atom parameters constrained
*wR*(*F*^2^) = 0.169	*w* = 1/[σ^2^(*F*_o_^2^) + (0.1443*P*)^2^ + 0.1456*P*] where *P* = (*F*_o_^2^ + 2*F*_c_^2^)/3
*S* = 1.09	(Δ/σ)_max_ < 0.001
839 reflections	Δρ_max_ = 0.80 e Å^−^^3^
81 parameters	Δρ_min_ = −0.82 e Å^−^^3^
0 restraints	Extinction correction: *SHELXL97* (Sheldrick, 2008), Fc^*^=kFc[1+0.001xFc^2^λ^3^/sin(2θ)]^-1/4^
Primary atom site location: structure-invariant direct methods	Extinction coefficient: 0.034 (9)

### Special details

**Table:**

Geometry. All e.s.d.'s (except the e.s.d. in the dihedral angle between two l.s. planes) are estimated using the full covariance matrix. The cell e.s.d.'s are taken into account individually in the estimation of e.s.d.'s in distances, angles and torsion angles; correlations between e.s.d.'s in cell parameters are only used when they are defined by crystal symmetry. An approximate (isotropic) treatment of cell e.s.d.'s is used for estimating e.s.d.'s involving l.s. planes.
Refinement. Refinement of *F*^2^ against ALL reflections. The weighted *R*-factor *wR* and goodness of fit *S* are based on *F*^2^, conventional *R*-factors *R* are based on *F*, with *F* set to zero for negative *F*^2^. The threshold expression of *F*^2^ > σ(*F*^2^) is used only for calculating *R*-factors(gt) *etc*. and is not relevant to the choice of reflections for refinement. *R*-factors based on *F*^2^ are statistically about twice as large as those based on *F*, and *R*- factors based on ALL data will be even larger.

### Fractional atomic coordinates and isotropic or equivalent isotropic displacement parameters (Å^2^)

**Table:**

	*x*	*y*	*z*	*U*_iso_*/*U*_eq_	
Zn1	0.15869 (6)	0.7500	0.0000	0.0503 (6)	
O1	0.1498 (3)	0.5678 (2)	0.07184 (15)	0.0600 (8)	
O2	−0.0220 (3)	0.6367 (2)	−0.07091 (14)	0.0592 (8)	
O3	0.3580 (3)	0.6927 (3)	−0.08757 (15)	0.0599 (8)	
N1	0.5920 (4)	0.7617 (2)	−0.1613 (2)	0.0541 (9)	
C1	−0.0497 (4)	0.5204 (3)	−0.0417 (2)	0.0498 (9)	
C2	0.4708 (5)	0.7774 (4)	−0.1043 (3)	0.0559 (10)	
H2	0.4686	0.8590	−0.0737	0.067*	
C3	0.6058 (5)	0.6357 (4)	−0.2119 (2)	0.0687 (11)	
H3A	0.6896	0.5769	−0.1858	0.103*	
H3B	0.6403	0.6571	−0.2701	0.103*	
H3C	0.4966	0.5905	−0.2130	0.103*	
C4	0.7261 (5)	0.8631 (4)	−0.1751 (3)	0.0741 (11)	
H4A	0.7063	0.9400	−0.1379	0.111*	
H4B	0.7244	0.8924	−0.2345	0.111*	
H4C	0.8358	0.8237	−0.1620	0.111*	

### Atomic displacement parameters (Å^2^)

**Table:**

	*U*^11^	*U*^22^	*U*^33^	*U*^12^	*U*^13^	*U*^23^
Zn1	0.0436 (8)	0.0500 (8)	0.0573 (8)	0.000	0.000	0.00088 (15)
O1	0.0572 (14)	0.0566 (14)	0.0661 (16)	−0.0077 (9)	−0.0120 (9)	0.0045 (10)
O2	0.0611 (15)	0.0539 (14)	0.0626 (15)	−0.0058 (9)	−0.0082 (9)	0.0082 (9)
O3	0.0538 (16)	0.0587 (17)	0.0672 (16)	−0.0041 (10)	0.0109 (9)	−0.0060 (12)
N1	0.0432 (19)	0.0546 (18)	0.065 (2)	0.0040 (10)	0.0055 (18)	0.0000 (10)
C1	0.0438 (14)	0.0507 (17)	0.055 (2)	0.0013 (12)	0.0012 (15)	0.0008 (13)
C2	0.051 (2)	0.0531 (17)	0.063 (2)	0.0038 (16)	−0.0031 (17)	−0.0021 (16)
C3	0.060 (2)	0.074 (2)	0.072 (2)	0.0038 (16)	0.0104 (18)	−0.0101 (17)
C4	0.057 (2)	0.065 (2)	0.101 (3)	−0.0029 (15)	0.014 (2)	0.0077 (18)

### Geometric parameters (Å, °)

**Table:**

Zn1—O2	2.101 (2)	N1—C3	1.466 (4)
Zn1—O2^i^	2.101 (2)	C1—O1^ii^	1.254 (4)
Zn1—O1	2.104 (2)	C1—C1^ii^	1.554 (6)
Zn1—O1^i^	2.104 (2)	C2—H2	0.9300
Zn1—O3^i^	2.134 (2)	C3—H3A	0.9600
Zn1—O3	2.134 (2)	C3—H3B	0.9600
O1—C1^ii^	1.254 (4)	C3—H3C	0.9600
O2—C1	1.246 (3)	C4—H4A	0.9600
O3—C2	1.237 (5)	C4—H4B	0.9600
N1—C2	1.299 (5)	C4—H4C	0.9600
N1—C4	1.458 (5)		
			
O2—Zn1—O2^i^	95.82 (14)	C4—N1—C3	116.4 (3)
O2—Zn1—O1	78.62 (8)	O2—C1—O1^ii^	127.3 (3)
O2^i^—Zn1—O1	98.81 (9)	O2—C1—C1^ii^	116.7 (3)
O2—Zn1—O1^i^	98.81 (9)	O1^ii^—C1—C1^ii^	116.1 (3)
O2^i^—Zn1—O1^i^	78.62 (8)	O3—C2—N1	125.3 (4)
O1—Zn1—O1^i^	176.23 (11)	O3—C2—H2	117.3
O2—Zn1—O3^i^	163.08 (9)	N1—C2—H2	117.3
O2^i^—Zn1—O3^i^	91.12 (9)	N1—C3—H3A	109.5
O1—Zn1—O3^i^	85.09 (9)	N1—C3—H3B	109.5
O1^i^—Zn1—O3^i^	97.67 (9)	H3A—C3—H3B	109.5
O2—Zn1—O3	91.12 (9)	N1—C3—H3C	109.5
O2^i^—Zn1—O3	163.08 (9)	H3A—C3—H3C	109.5
O1—Zn1—O3	97.67 (9)	H3B—C3—H3C	109.5
O1^i^—Zn1—O3	85.09 (9)	N1—C4—H4A	109.5
O3^i^—Zn1—O3	86.53 (13)	N1—C4—H4B	109.5
C1^ii^—O1—Zn1	114.3 (2)	H4A—C4—H4B	109.5
C1—O2—Zn1	114.36 (19)	N1—C4—H4C	109.5
C2—O3—Zn1	118.2 (2)	H4A—C4—H4C	109.5
C2—N1—C4	122.6 (3)	H4B—C4—H4C	109.5
C2—N1—C3	120.9 (3)		
			
O2—Zn1—O1—C1^ii^	0.7 (2)	O2^i^—Zn1—O3—C2	29.7 (5)
O2^i^—Zn1—O1—C1^ii^	94.9 (2)	O1—Zn1—O3—C2	−137.2 (3)
O3^i^—Zn1—O1—C1^ii^	−174.7 (2)	O1^i^—Zn1—O3—C2	45.3 (3)
O3—Zn1—O1—C1^ii^	−88.9 (2)	O3^i^—Zn1—O3—C2	−52.7 (2)
O2^i^—Zn1—O2—C1	−98.8 (2)	Zn1—O2—C1—O1^ii^	−179.4 (3)
O1—Zn1—O2—C1	−0.9 (2)	Zn1—O2—C1—C1^ii^	0.9 (4)
O1^i^—Zn1—O2—C1	−178.1 (2)	Zn1—O3—C2—N1	−174.2 (3)
O3^i^—Zn1—O2—C1	15.0 (4)	C4—N1—C2—O3	−177.0 (4)
O3—Zn1—O2—C1	96.7 (2)	C3—N1—C2—O3	−0.6 (6)
O2—Zn1—O3—C2	144.1 (3)		

Symmetry codes: (i) *x*, −*y*+3/2, −*z*; (ii) −*x*, −*y*+1, −*z*.
